# Spatiotemporal Distribution of U5MR and Their Relationship with Geographic and Socioeconomic Factors in China

**DOI:** 10.3390/ijerph14111428

**Published:** 2017-11-21

**Authors:** Zeng Li, Jingying Fu, Dong Jiang, Gang Lin, Donglin Dong, Xiaoxi Yan

**Affiliations:** 1 College of Geoscience and Surveying Engineering, China University of Mining & Technology (Beijing), Ding No.11 Xueyuan Road, Haidian District, Beijing 100083, China; lizeng_cumtb@163.com (Z.L.); ddl9266@163.com (D.D.); 2 Institute of Geographical Sciences and Natural Resources Research, Chinese Academy of Sciences, 11A Datun Road, Chaoyang District, Beijing 100101, China; fujy@igsnrr.ac.cn (J.F.); yanxx.15s@igsnrr.ac.cn (X.Y.); 3 College of Resource and Environment, University of Chinese Academy of Sciences, No. 19A Yuquan Road, Beijing 100049, China

**Keywords:** U5MR, nighttime lights, digital elevation model, GWR

## Abstract

Epidemiological studies conducted around the world have reported that the under-five mortality rate (U5MR) is closely associated with income and educational attainment. However, geographic elements should also remain a major concern in further improving child health issues, since they often play an important role in the survival environment. This study was undertaken to investigate the relationship between the U5MR, geographic, and socioeconomic factors, and to explore the associated spatial variance of the relationship in China using the geographically weighted regression (GWR) model. The results indicate that the space pattern of a high U5MR had been narrowed notably during the period from 2001 to 2010. Nighttime lights (NL) and the digital elevation model (DEM) both have obvious influences on the U5MR, with the NL having a negative impact and DEM having a positive impact. Additionally, the relationship between the NL and DEM varied over space in China. Moreover, the relevance between U5MR and DEM was narrowed in 2010 compared to 2001, which indicates that the development of economic and medical standards can overcome geographical limits.

## 1. Introduction

The under-five mortality rate (U5MR) is expressed as the probability of dying before five years of age per 1000 newborns [1]. U5MR is an indicator used to monitor child survival and is also an important reflection of the economy, society, and environment [2]. As one of the key indicators, it is included in Millennium Development Goal 4 (MDG 4) [3] and is mentioned again in the Sustainable Development Goals (SDG) by 2030, with all countries aiming to reduce the neonatal U5MR to no more than 25% of live births [4,5]. With the increase of the level of medical and health causes, this effort has contributed to a 50% decrease in child deaths worldwide between 2010 and 2015, but an astounding 16,000 children under five years die every day due to diseases that are mostly preventable [6]. Additionally, it is also reported that there was a significant variation of the U5MR in space, and the survival rate of children has a close relationship with the birthplace [6]. For example, there may be one child that died for every 12 persons in Sub-Saharan Africa, which is almost 12 times more than in affluent regions. This U5MR is the highest in the world, even though significant advances have been made in reducing this death rate [7]. Over several decades, China has made tremendous progress in reducing the U5MR. China researched the MDG 4 target of U5MR, reducing the U5MR 7 years in advance of the target date in 2008 [8,9]. The U5MR has fallen to 10.7‰ in 2015, which also achieved the SDG target [10]. However, it is noteworthy that there are still many children that have died per year under a giant base population. Worse, large gaps remain in the U5MR between East and West China, and disparities also exist between rural and urban residents. The U5MR in China varies from different provinces and regions [8]. It is essential to evaluate the influences of geographical elements on the U5MR in China to improve the decision-making skills of their public health program.

In recent years, increasing the number of studies examining the associations between the U5MR and economic and social factors have been published, with most of the studies reporting that the national success in reducing child mortality is attributed to economic growth and advisable health policies, as well as the improvement of female education and empowerment [11,12,13]. Wang et al. conducted a population-based survey to explore the mortality rate and the leading causes of death for children under five years of age and later reported that the U5MR would continue to decline due to rapid socioeconomic development, public affairs, and utilities [14]. In 2012, Feng et al. concluded that vertical intervention programs, strengthening of health systems or economic growth are significantly associated with child mortality in China, and the cross-sectoral approach might significantly contribute to the reduction of the U5MR [11]. Based on the study of the correlation between the U5MR and socioeconomic factors, recent studies have also been performed to estimate child mortality over an extended time series [8,13]. Furthermore, there have also been many previous studies reporting that the U5MR is often linked with environmental pollution. Geographical factors are also a good indicator of infant mortality, since climate and geography often play a role in their environment [15,16]. The studies conclude that regions that have higher particulate matter (PM) also have a greater chance of having a higher mortality rate, including infant mortality [17,18], and areas with carbon monoxide increase the infant mortality rates, as well [19]. Based on the reported U5MR information, Yang et al. investigated the relationship between the family geographic environment and the death of children of different genders under age 5 in Hubei Province, China and observed that the U5MR is closely related with the family geographic environment [20]. Therefore, it is important to understand the relationship between geographical elements and the U5MR in space, since different regions have different environmental and socioeconomic characteristics. However, due to the lack of the refined long-term U5MR data and the gridded products for geographical and socioeconomic factors, studies on the spatial relationship between them have seldom been conducted in China.

The objective of this paper is to investigate the spatiotemporal pattern of U5MR. Next, we explore U5MR’s relationship with geographic and socioeconomic factors and the associated spatial variance of the relationship in China based on long-term spatial data. It is expected to guide both the central and local governments to form a better localized policy to reduce child mortality in regions that are still lagging behind and also to provide implications for policy makers outside of China. The estimated U5MR data, gridded nighttime lights (NL) data and gridded digital elevation model (DEM) for the period from 2001 to 2010 were used in the analysis. The influences of the geographic and socioeconomic factors in relation to U5MR in space were analyzed using the geographically weighted regression (GWR) model.

## 2. Data Acquisition

### 2.1. U5MR Data

**Figure 1:**
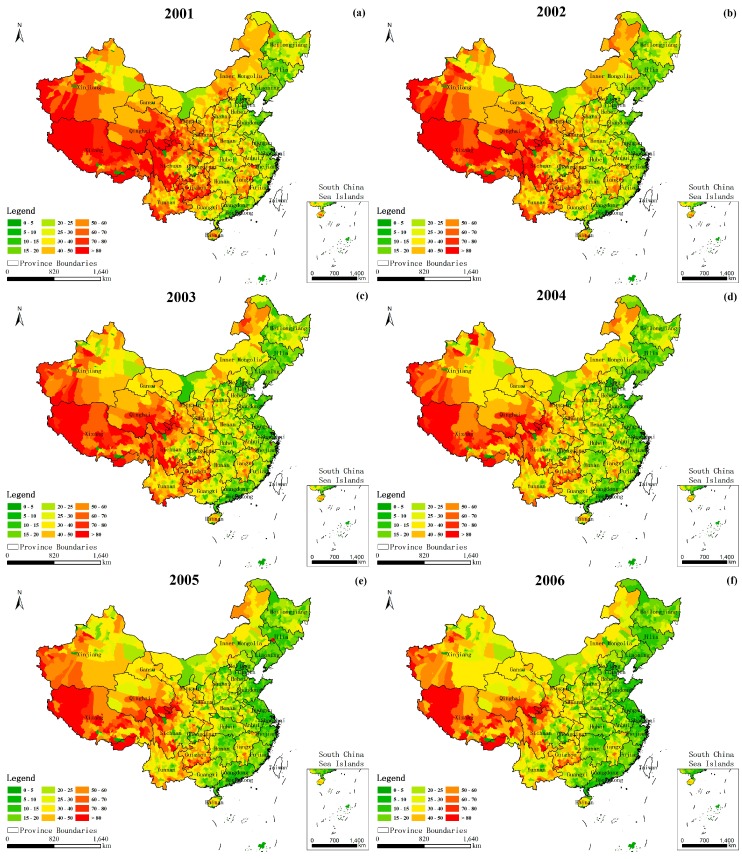

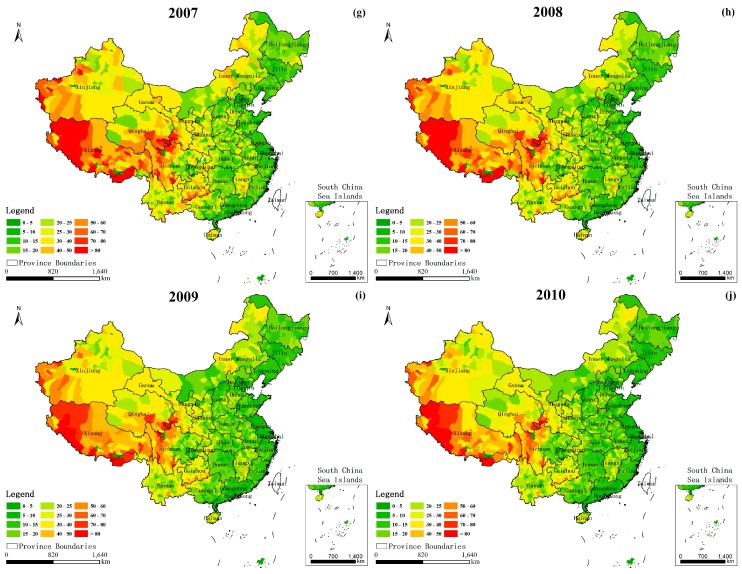
Spatial distribution of the U5MR in China from 2001 to 2010 (**a**–**j**).

The estimated child mortality dataset in the 2851 counties in China has never been provided until they were published by Wang et al. in 2015 [8]. Using several surveys, surveillance systems, censuses, and vital registration sources of data for child mortality, the U5MR dataset was the first publication to present a careful quantification of the levels and trends in child mortality at the county level in China from 1996 to 2012 [8]. Those systematic document levels and trends for U5MR at the county level can help to form better public health strategies and conduct further epidemiological studies. In this study, the data from 2001 to 2010 was selected considering the time matching to the geographic and socioeconomic gridded data. Figure 1a–j shows the spatial distribution of U5MR in China from 2001 to 2010.

### 2.2. NL Data

**Figure 2:**
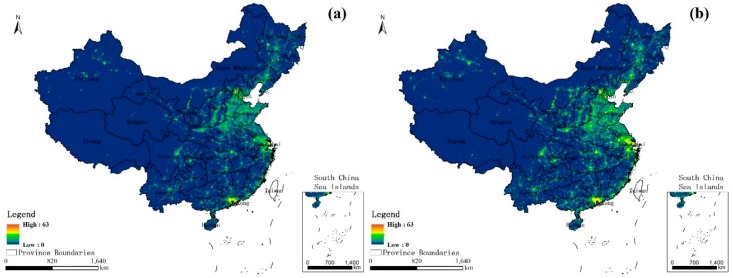
Spatial distribution of the nighttime light image in 2001 (**a**) and 2010 (**b**) in China.

NL data is well-suited to large-scale monitoring research, since it can express the spatial information and intensity change which is always used to reflect the socioeconomic characteristics. A large number of previous studies have demonstrated that nightlights can be considered a good developmental indicator for the estimation of the Gross Domestic Product (GDP) [21], population density [22,23], energy consumption [24], and urban sprawl [25], and is often exploited to derive the global mapping of socioeconomic parameters [26]. Thus, in this study, the NL dataset was selected as a comprehensive index, which could reflect the spatiotemporal characteristics of the socioeconomic characteristics from 2001 to 2010, which were obtained from NOAA’s National Centers for Environmental Information (NCEI) [27]. Figure 2 shows the spatial distribution of the nighttime light image in 2001 (a) and 2010 (b) in China.

### 2.3. DEM Data

**Figure 3:**
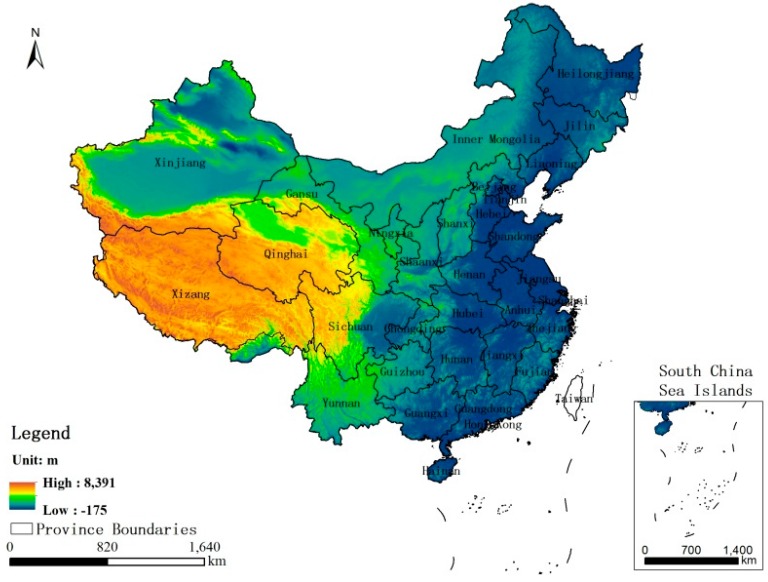
Spatial distribution of the DEM in China.

The DEM data are an important source of original data for the studies of terrain, watershed, and ground feature recognition. These data describe the height information and has wide applications in the sciences, humanities, and social sciences [28,29,30,31,32]. The gridded data (approx. 90 m resolution) of this study used to be a geographical element for building the GWR model and was extracted from the SRTM digital elevation product originally produced by the National Aeronautics and Space Administration (NASA). Moreover, considering that there has not been notable change in DEM for 10 years, the same data was applied for both the 2001 and 2010 GWR model. Figure 3 shows the DEM in China.

## 3. Methodology

The spatiotemporal distribution of U5MR and their relationship with geographic and socioeconomic factors were evaluated using the following steps:

Step 1: Evaluate the spatial variations of U5MR in China from 2001 to 2010 and the cluster feature by the spatial autocorrelation model. The impact of the GDP on the U5MR was also analyzed;

Step 2: Before exploring the spatial variation of the relationship between the U5MR and geographic and the socioeconomic factors, the Pearson’s correlation method was performed to select the variable factors for the spatial model; and

Step 3: Use the GWR method to evaluate the relationships between the U5MR, the NL and the DEM and also investigate the associated spatial variance of the relationship in China based on the raster data.

### 3.1. Spatial Autocorrelation Model

Before the GWR model can occur, Global Moran’s I, a common index to measure spatial autocorrelation, was used to determine the spatial pattern of U5MR by the spatial autocorrelation model [33]. This index can detect spatial disparity, which is induced by the local social economic development level and structure between samples [34]. Positive spatial autocorrelation appeared when Moran’s I values were larger than zero. The negative spatial autocorrelation is denoted when the values are smaller than zero, and no spatial autocorrelation existed when the values were near zero [35]. Moran’s I can be defined using the following equation [36]:

(1) $$I=\frac{n}{\sum_{i}^{n}\sum_{j}^{n}w_{ij}}\times \frac{\sum_{i}^{n}\sum_{j}^{n}w_{ij}\left(r_{i}-\bar{r}\right)\left(r_{j}-\bar{r}\right)}{\sum_{i}^{n}{\left(r_{i}-\bar{r}\right)}^{2}}$$

where n stands for the sum total of the samples; $w_{ij}$ is the spatial weight between point i and point j; $r_{i}$ and $r_{j}$ are the values of U5MR for the *i*th and *j*th points, respectively; and $\bar{r}$ is the mean value [36].

### 3.2. GWR Model

For this epidemiology study, the GWR model was used to estimate the relation between the U5MR and the geographic and socioeconomic factors. The GWR model is developed to explore the spatial heterogeneity by insetting the spatial locations of the regression parameters in the regression model [37]. This model considers the local estimates of the parameters by building a local regression model and investigates the impact from the independent variables on the dependent variables with the changes in locations [38]. The spatial heterogeneity of the relationship between an independent variable and dependent variable can be well-expressed using the model [39]. This model has been extensively used for epidemiology in recent publications [17,40], hence, a much more detailed summary is not provided here. The model was adopted in this paper to indicate the spatial variance of the relationship between U5MR and the geographic and socioeconomic factors. The model can be expressed using the following formula:

(2) $$y_{i}=\delta_{0}\left(\alpha_{i},\beta_{i}\right)+\underset{k}{\sum }\delta_{k}\left(\alpha_{i},\beta_{i}\right)x_{ik}+\theta_{i},\;\left(i=1,2,\ldots ,n\right)$$

where $\left(\alpha_{i},\beta_{i}\right)$ stands for the spatial coordinate of sample point i and where $\delta_{k}\left(\alpha_{i},\beta_{i}\right)$ is the regression coefficient of sample point i. $\theta_{i}$ is the random error of the independent distribution, which is usually assumed to obey a normal distribution. In the present model of this study, we chose an adaptive kernel with a bandwidth (an important parameter for the GWR model to control the degree of smoothing) that was decided by minimizing the corrected Akaike information criterion (AIC) by evaluating the spatial configuration of the features [37,38].

## 4. Results and Analysis

### 4.1. Spatiotemporal Pattern of U5MR Change Analysis

**Figure 4:**
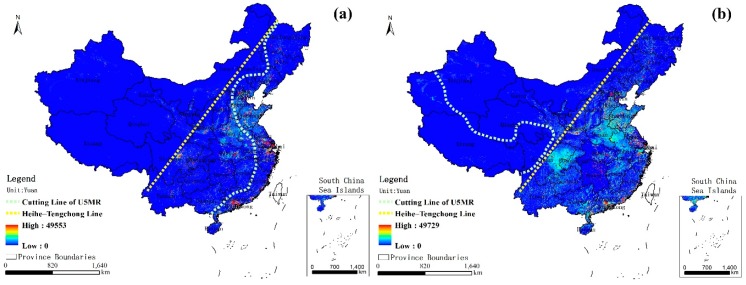
GDP distribution in China for (**a**) 2001 and (**b**) 2010. Note: the yellow line is the “Heihe-Tengchong Line”, and the green line is the cutting line between the high U5MR values and the low U5MR values. The U5MR in the areas of the left of the green line are almost beyond 40/1000 (Figure 1).

**Figure 5:**
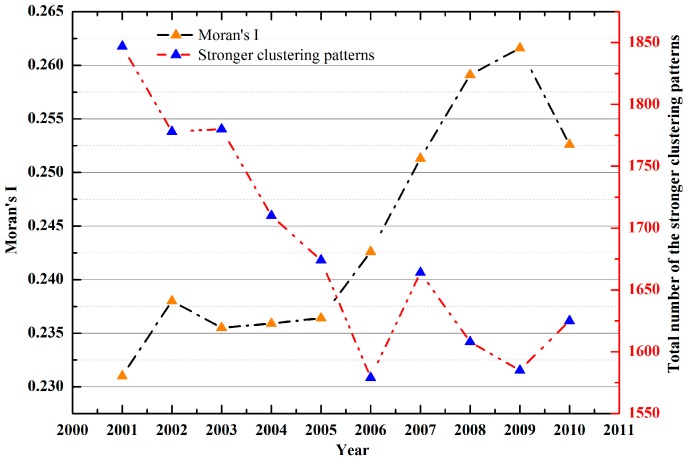
Moran’s I of the spatial autocorrelation model for U5MR from 2001 to 2010 (Y-axis in the left) and the total number of the stronger clustering patterns (Y-axis in the right). Note: the Z-values are all greater than 1.96 and the *p*-values are all less than 0.01.

Figure 4 shows the GDP distribution in China for (a) 2001 and (b) 2010. The Heihe-Tengchong Line (the yellow line in Figure 4) is a classical geographical boundary which divides China into two roughly equal parts, clearly recording the pattern of development of the economic society [41,42]. In the eastern region denoted by the line, the level of urbanization is higher than in the western areas during the past ten years, except that there has been a small amount of development in the early regions of Western China in 2010, such as in the Gansu, Ningxia, and Qinghai provinces. As always, one reason for this is the two rather different natural geographical environments between the two parts. Arid and half-arid desert mine areas and snow-covered plateaus are widely observed in the western region, whereas plain water system areas are widely distributed in the eastern region. Seen from the figure, the green line cuts between the high U5MR values and the low values from 2001 and 2010, and the U5MR to the left of the green line is almost beyond 40/1000. From the geographical pattern, the nation has been highly successful in reducing child mortality. The U5MR beyond 40/1000 spreads all over the country in 2001 but narrows to only a small part of the southwestern region in 2010, which are mostly the desert and plateau regions, such as in most of Xizang and a fraction of Xinjiang, Qinghai, and Sichuan Provinces. In most of the previous empirical studies, there is a view that the success in reducing the U5MR in China is primarily attributable to the economic growth and advisable health policies, as well as the improvement of female education and empowerment [11,12,13]. From a distance, the economic factors, to a certain extent, are important contributors in reducing the U5MR, but there are also differences between them in the evolution model. Due to the geographical factors and historical factors, the U5MR of the country is regionally unbalanced (Figure 1). However, there are few studies reporting the relationship between the geographical elements and the U5MR in space, especially for the nation. Figure 5 shows the Moran’s I of the spatial autocorrelation model for the U5MR from 2001 to 2010. The Moran’s I values are all larger than zero over the ten years, which indicates that they have significant spatial clustering characteristics. Thus, this approach has significant value for exploring the influence of the geographic and socioeconomic factors on the U5MR in space for the nation.

### 4.2. Spatial Variation of the Identified Relationship

Night lights have been proven to be a good development indicator for GDP, population density, energy consumption, and urban sprawl, and it can be considered a composite index for economic and social development in China [21,22,23,24,25]. Thus, the NL dataset was selected in this study to represent the spatiotemporal characteristics of the socioeconomic characteristics for the GWR model. Regarding the geographic factors, the Pearson’s correlation method was used to select the primary affective factors. Table 1 shows the Pearson correlation between the U5MR and the geographic factors (temperature, precipitation and DEM, here) from 2001 to 2010. As can be seen from the table, the mean value of the Pearson’s for temperature and precipitation are −0.168 and −0.130, respectively, which indicates that they have a poor correlation with the U5MR in China from 2001 to 2010. In contrast, from the Pearson model, we observed that there is an obvious positive correlation between the U5MR and the DEM with a mean Pearson’s *r* value of 0.628, which is statistically significant. Therefore, in this study we selected the DEM model as a geographical influencing variable for the GWR model.

The adjusted R^2^ of the GWR models for the relationship between the U5MR with geographic and socioeconomic factors was 0.740, which indicates that the fitted effect of the model was better and that the U5MR has higher relevance with the NL and DEM. The condition numbers of the model, which were used for detecting the existence of the collinearity problem, were all less than 15, indicating weak dependencies in the data [38]. Based on the method used in the previous studies [43,44], the f-statistic was used to explore the spatial instability of the coefficients (it is calibrated using GWR 4.0 with the Leung test calculating the *f* = *V_GWR_*/*RSS_GWR_* (where *V_GWR_* is the variance of the coefficients and *RSS_GWR_* is the residual sum of squares)), and the results of the F-test for the model suggested statistical significance (*p* < 0.05), which shows the non-stationary spatial variation of the regression coefficients.

**Figure 6:**
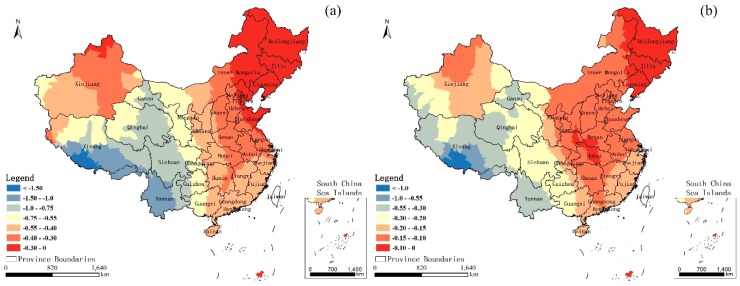
Local coefficients of the NL for (**a**) 2001 and (**b**) 2010.

**Figure 7:**
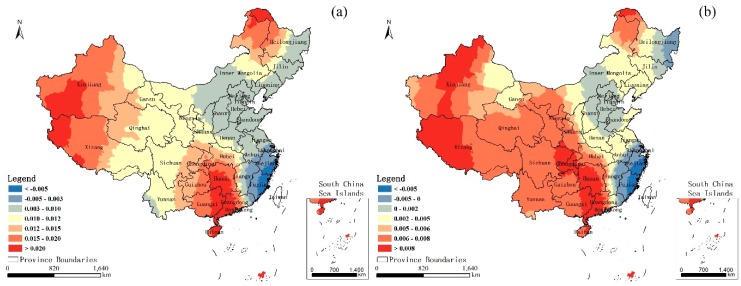
Local DEM coefficients for (**a**) 2001 and (**b**) 2010.

Figure 6 shows the local NL coefficients for (a) 2001 and (b) 2010. In general, the local NL coefficients in 2001 indicate that their influence on the U5MR varied considerably over the entire country and the whole study area demonstrated a negative correlation between the U5MR and NL. In general, the correlation successively increased from northeast to southwest, and the strong correlations with the high coefficients mainly appeared in the southwest provinces of the country, such as Yunnan, Xizang, Sichuan, and Qinghai. In 2010, the overall map is similar to that in 2001. The correlation also increases from the northeast to the southwest in space, but the overall relevancy decreases to a certain extent compared to 2001. The strong correlations in 2010 mainly appeared in the Xizang and Yunnan Provinces. Figure 7 shows the local DEM coefficients for (a) 2001 and (b) 2010. Unlike the NL situation, most of the study area demonstrated a positive correlation between the U5MR and DEM in both 2001 and 2010, which indicates that high altitude has a promoting effect on the U5MR. Overall, the correlations in the regions with the higher development level are smaller than the relatively poor areas in both years, and the relevancy decreases to a certain extent in 2010 compared to 2001. In addition, the level of the social economy in 2010 China has rapidly improved compared to 2001. Thus, we deduced that the cause of the reduction of the correlations between the U5MR and DEM was mainly because of the rapid development of the social economy and, to a certain degree, the rapid development of the social economy can overcome the geography limits impacting the U5MR.

## 5. Discussion

In certain previous studies, the economic and social factors, such as income and educational attainment, have been considered to be important variables for the estimation of the U5MR [8,13], but the geographic elements have remained a major concern. From the GWR model, regions with StdResid values (the standardized residual) in the range of −2.5 to 2.5 composed 97.5% in 2001 and 98.2% in 2010 of the entire country. The spatial autocorrelation model was also run for the residuals to make sure that they are spatially random. Additionally, the higher adjusted R^2^ of the GWR models, it is thereby that most of China has exhibited better predictive ability of the DEM for U5MR. Thus, combined with the socioeconomic factors, the geographic elements are expected to be good predictive variables for estimating the U5MR, especially from the perspective in the geometric space.

## 6. Conclusions

This paper explores the spatiotemporal pattern of the U5MR from 2001 to 2010 and investigates the influence from the geographic and socioeconomic factors and the associated spatial variance of the relationship in China using the explicit spatial data of the NL and DEM. The following primary conclusions were attained:

(1) The space pattern of the U5MR was reduced tremendously from 2001 to 2010. The regions with values more than 40/1000 have been narrowed to the Tibetan Plateau regions.

(2) In general, the relationship between the U5MR, the NL and the DEM varied over space for both 2001 and 2010. There has been a positive correlation between the U5MR and the DEM, while there has been a negative correlation between U5MR and the NL. After ten years, the relevance between the U5MR and the DEM was narrowed in 2010, meaning, to a certain degree, that the development of the economic and medical standards can overcome the geographical limitations. However, these factors remain important for the estimation of the U5MR.

This study provides a valuable epidemiological reference in further improving child health. However, the problem is complex, and further analysis, especially from the spatial perspective, is warranted.

## Figures and Tables

**Table 1:** Pearson’s *r* between the U5MR and the geographic factors (N = 2886).

Items	Temperature		Precipitation		DEM	
	*r*	*p*-Value	*r*	*p*-Value	*r*	*p*-Value
2001	0.190	0.000	0.107	0.000	0.660	0.000
2002	0.004	0.416	0.012	0.063	0.629	0.000
2003	0.091	0.000	0.104	0.000	0.673	0.000
2004	0.091	0.000	0.070	0.000	0.568	0.000
2005	0.265	0.000	0.205	0.000	0.667	0.000
2006	0.283	0.000	0.208	0.000	0.677	0.000
2007	0.002	0.045	0.014	0.221	0.612	0.000
2008	0.184	0.000	0.123	0.000	0.406	0.000
2009	0.290	0.000	0.254	0.000	0.699	0.000
2010	0.280	0.000	0.222	0.000	0.688	0.000
